# Adaptive Slicing Method of the Spatiotemporal Event Stream Obtained from a Dynamic Vision Sensor

**DOI:** 10.3390/s22072614

**Published:** 2022-03-29

**Authors:** Yisa Zhang, Yuchen Zhao, Hengyi Lv, Yang Feng, Hailong Liu, Chengshan Han

**Affiliations:** 1 Changchun Institute of Optics, Fine Mechanics and Physics, Chinese Academy of Sciences, Changchun 130033, China; zhangyisa18@mails.ucas.ac.cn (Y.Z.); bernard19@163.com (Y.Z.); fenyang16@mails.ucas.edu.cn (Y.F.); ustclhl@163.com (H.L.); hancs@ciomp.ac.cn (C.H.); 2 College of Materials Science and Opto-Electronic Technology, University of Chinese Academy of Sciences, Beijing 100049, China

**Keywords:** dynamic vision sensor, spatiotemporal event stream, adaptive slicing

## Abstract

The dynamic vision sensor (DVS) measures asynchronously change of brightness per pixel, then outputs an asynchronous and discrete stream of spatiotemporal event information that encodes the time, location, and sign of brightness changes. The dynamic vision sensor has outstanding properties compared to sensors of traditional cameras, with very high dynamic range, high temporal resolution, low power consumption, and does not suffer from motion blur. Hence, dynamic vision sensors have considerable potential for computer vision in scenarios that are challenging for traditional cameras. However, the spatiotemporal event stream has low visualization and is incompatible with existing image processing algorithms. In order to solve this problem, this paper proposes a new adaptive slicing method for the spatiotemporal event stream. The resulting slices of the spatiotemporal event stream contain complete object information, with no motion blur. The slices can be processed either with event-based algorithms or by constructing slices into virtual frames and processing them with traditional image processing algorithms. We tested our slicing method using public as well as our own data sets. The difference between the object information entropy of the slice and the ideal object information entropy is less than 1%.

## 1. Introduction

Currently, the mainstream imaging devices are CCD and CMOS image sensors, which output relatively intuitive and eye-pleasing images at a fixed frame rate. However, the frame-based sensor captures a series of frames that have information about the entire scene in the field of view. Therefore, there is a lot of redundant background information in each frame when we apply a camera to object tracking. Moreover, due to the limitation of frame rate, the motion information of high-speed moving objects will be lost between frames. In order to meet the needs of computer vision in challenging scenarios for frame-based cameras, people invented dynamic vision sensors [1,2,3,4]. As a result of their unique pixel structure, these sensors only respond where the light intensity changes, and have the advantages of high dynamic range, low data volume, and low power consumption [5]. Hence, dynamic vision sensors have been gradually applied to object tracking [6,7,8], surveillance and monitoring [9,10,11,12,13], star tracking [14], etc.

### 1.1. Dynamic Vision Sensor

**Figure 1:**
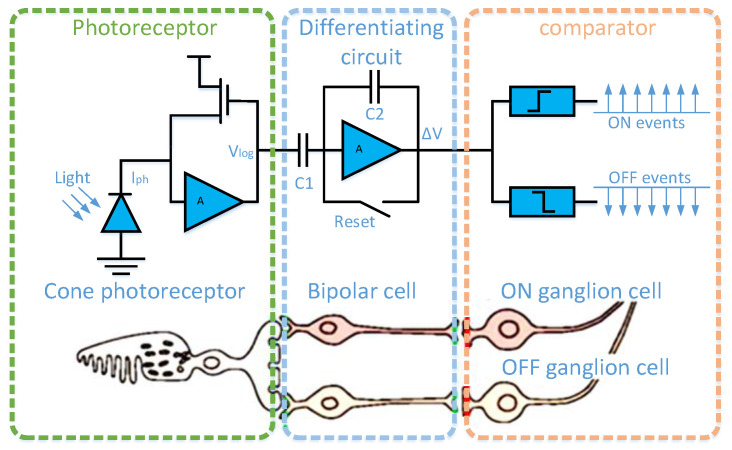
Three-layer model of a human retina and corresponding DVS pixel circuitry. The first layer is similar to retinal cone cells for photoelectric conversion; the second layer, similar to bipolar cells in the retina, is used to obtain changes in light intensity; the third layer is similar to the ganglion cells of the retina for outputting the light intensity change sign.

The dynamic vision sensor is inspired by the biological retina, and its structural composition is shown in Figure 1 [15]. A pixel of the dynamic vision sensor consists of a fast logarithmic photoreceptor, a differencing circuit, and two comparators. The fast logarithmic photoreceptor circuit is similar to the cone cells in the retina for photoelectric conversion. The differential circuit, like bipolar cells in the retina, is used to obtain changes in light intensity. The comparison circuit is similar to the retina’s ganglion cells for outputting the light intensity change sign. When the light intensity is enhanced, it outputs an ON signal; otherwise, it outputs an OFF signal. As a result of its unique pixel structure, its working principle is similar to the human channel attention mechanism, as the dynamic vision sensor only responds to places where the light intensity changes in the scene, hence there is no data redundancy. The output ON/OFF signal is called event $e_{i}=e\left(x_{i},y_{i},t_{i},p_{i}\right)$, which contains position, microsecond timestamp, and polarity information. Combined with the characteristics of the dynamic vision sensor, we call the set of output events in the spatiotemporal domain as the spatiotemporal event stream. The spatiotemporal event stream can be defined as the following:

(1) $$E=\overset{N}{\underset{i=1}{\sum }}e\left(x_{i},y_{i},t_{i},p_{i}\right)$$

where e is an event of the spatiotemporal event stream, [*x*, *y*] denotes location of the pixel generating the event, *p* ∈ {−1, +1} indicates the polarity of the change in illumination at the pixel causing the event, and *t* represents the time at which the event occurred; i is the index of events in the spatiotemporal event stream, and Σ indicates adding the new event to the spatiotemporal data stream.

### 1.2. The Related Work to Building Virtual Frames by Accumulating Events

**Figure 2:**
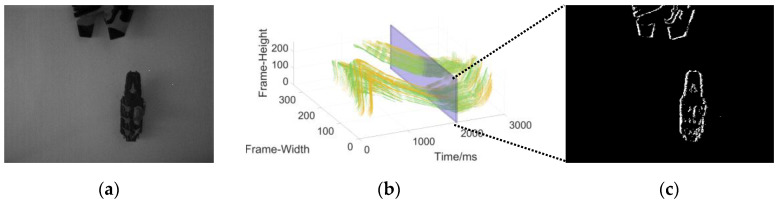
Illustration of DVS output and virtual frame. (**a**) Moving object of DVS observation; (**b**) the visualization of the event stream, DVS output is the event stream of address events (x,y) in time. Each address event signals that the pixel at that coordinate experienced a change of light at that instant. (**c**) The virtual frame constructed by the slice in (**b**).

DVS output is an event stream of address events (x,y) in time, hence the output data of the dynamic vision sensor are called the spatiotemporal event stream, as shown in Figure 2. As a result of changes in the speed or the number of objects, the event distribution will change sharply in space and time. Moreover, in the field of machine vision, most researchers slice the spatiotemporal data stream with a constant time interval or a constant event number [16,17,18,19,20] to construct a virtual frame, and then use traditional image processing methods for object recognition or tracking. In general, we think an good virtual frame should include complete object information and no motion blur, which is a good beginning for further image processing. On the contrary, if object information is missing or there is motion blur in the virtual frame, the object recognition accuracy and other image processing effects will be seriously affected. Therefore, it is essential to choose an appropriate event slicing method.

The constant time interval slice of the event stream is defined as follows:

(2) $$E\left(\tau \right)=\overset{t_{k}+\tau }{\underset{t_{k}}{\sum }}\{e\left(x_{i},y_{i},t_{i},p_{i}\right)|t_{i}\in \left[t_{k},t_{k}+\tau \right]\}\;$$

Therefore, the virtual frame obtained through the constant time interval is defined as follows:

(3) $$f\left(x,y,T\right)=\overset{t_{k}+\tau }{\underset{t_{k}}{\sum }}\{e\left(x_{i},y_{i},t_{i},p_{i}\right)|t_{i}\in \left[t_{k},t_{k}+\tau \right]\}$$

In the same way, the constant event number slice of the event stream and the constructed virtual frame are defined below as (4) and (5):

(4) $$E\left(N\right)=\overset{m+N}{\underset{m}{\sum }}\{e\left(x_{i},y_{i},t_{i},p_{i}\right)|i\in \left[m,m+N\right]\}$$

(5) $$f\left(x,y,T\right)=\overset{m+N}{\underset{m}{\sum }}\{e\left(x_{i},y_{i},t_{i},p_{i}\right)|i\in \left[m,m+N\right]\}$$

where τ and N are fixed values obtained by experience. T is the mean timestamp of all events in the event stream.

**Figure 3:**
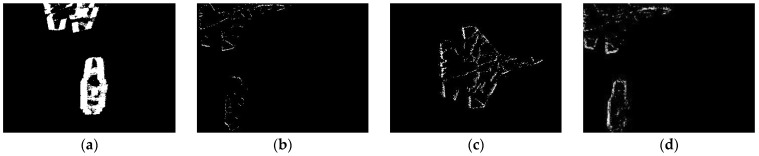

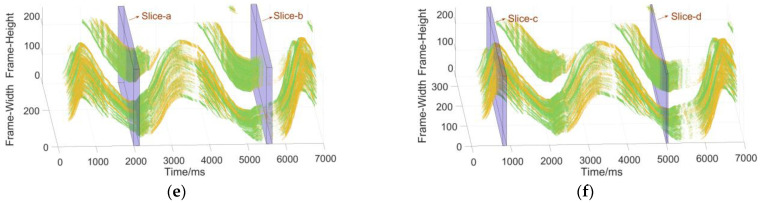
(**a**,**b**) are virtual frames constructed from slice-a and slice-b of (**e**), which are sliced by the constant time interval. The object in fast motion generates very dense events, hence the virtual frame (**a**) constructed by slice-a has motion blur; the object in slow motion generates very sparse events, thus the virtual frame (**b**) constructed by slice-b loses object information. (**c**,**d**) are virtual frames constructed from slice-c and slice-d of (**f**), which are sliced by the constant event number. Due to the change of speed or number of objects, the number of events in the event flow changes sharply. Therefore, the number of events in slice-c is not suitable for slice-d; the virtual frame (**c**) constructed by slice-c has neither motion blur nor information loss, but the virtual frame (**d**) constructed by slice-d loses object information. (**e**,**f**) are spatiotemporal distributions of event streams caused by moving objects. As a result of the change of the speed and number of objects, the event distribution changes sharply in space and time.

Although the two slicing methods of the constant time interval and constant event number are simple and direct, the slicing effect is limited by the object speed and number, and the slicing quality is not ideal for dynamic scenes. When the object speed or number changes, if the time interval is too long or the number of events is too large, this will result in motion blur, as shown in Figure 3a. On the contrary, if the time interval is too short or the number of events is too small, the object information will be lost, as shown in Figure 3b,d. The motion blur and object information loss will bring computational errors to object recognition and tracking, making it difficult to artificially determine the time interval or the number of events.

The Adaptive Time-Surface with Linear Time Decay (ATSLTD) event-to-frame conversion algorithm in [21] slices the spatiotemporal event stream by calculating the confidence interval of the information entropy of the virtual frames with sharp and clear edges. However, the confidence interval of the information entropy has not been updated, thus it is not suitable for complex motion scenes. The authors of [22] propose a method called AreaEventNumber; instead of rotating the slices based on the sum of the whole slice event number, AreaEventNumber triggers the slice rotation once any one of the area’s event numbers (Area Event Counters) exceeds the threshold value k. However, this method still requires experience to determine the threshold k.

In order to solve the problem of motion blur or object information loss caused by improper slicing of the spatiotemporal event stream, we propose a new adaptive slicing method for the spatiotemporal event stream. The event slice is defined as follows:

(6) $$E\left(\Delta t\right)=\overset{t_{k}+\Delta t}{\underset{t_{k}}{\sum }}\{e\left(x_{i},y_{i},t_{i},p_{i}\right)|t_{i}\in \left[t_{k},t_{k}+\Delta t\right]\}$$

where $t_{k}$ is the start of the event slice, Δt represents the time length of the event slice, and E(Δt) represents the event slice.

**Figure 4:**
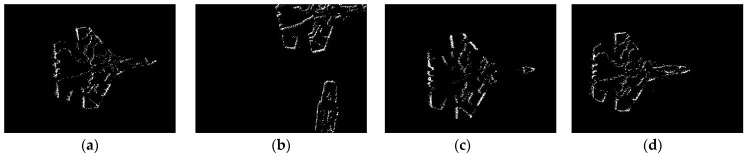

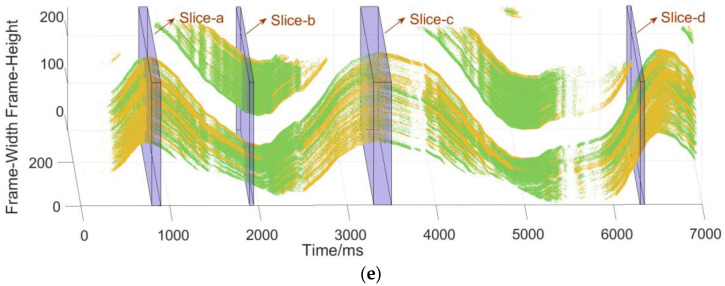
(**a**–**d**) are virtual frames constructed by adaptive slicing from the spatiotemporal event stream (**e**). When the number of objects changes or the speed of objects changes, our method can select appropriate slices from the rapidly changing event stream to construct a virtual frame without motion blur and information loss; (**e**) the spatiotemporal distribution of event stream caused by moving object.

When the speed or the number of objects changes, Δt also adjusts dynamically, and there is no motion blur or information loss in E(Δt). The slicing effect is shown in Figure 4.

### 1.3. The Main Contributions of This Paper

Firstly, this paper proposes a past events remove mechanism to obtain a reference frame with clear and sharp edges while reducing noise; secondly, a group of virtual frames $F\left(n\right)=\{f_{1},f_{2},\ldots f_{n}\}$ is randomly constructed from event slices to calculate the similarity $S_{n}=\left\{s_{1},s_{2},\ldots s_{n}\right\}$ between F(n) and the reference frame, and then use $S_{n}$ to calculate the confidence interval. Finally, the events are accumulated in millisecond units to construct the virtual frame until the similarity between the virtual frame and the reference frame is within the confidence interval. The confidence interval is updated dynamically with the scene change. In a word, the main contributions of this paper are as follows:

(1) A past event elimination mechanism is proposed, which can obtain a virtual frame with clear and sharp edges at any time;
(2) The adaptive slicing of the spatiotemporal event stream will not cause object motion blur or loss of object information;
(3) In order to adapt to different motion scenes, the calculation parameters are updated adaptively.

## 2. Materials and Methods

In this part, we first explain how to use the past events remove mechanism to obtain an ideal frame with clear and sharp edges in Section 2.1, and then introduce a method to adaptively slice the spatiotemporal event stream to ensure that the spatiotemporal event slice contains complete object information without motion blur in Section 2.2. Next, we introduce our method in detail.

### 2.1. The Past Events Remove Mechanism

This method is inspired by the authors of [23] who use a local planar approximation of the surface of active events to calculate the lifetime of events, then use the new event’s velocity information to reset the lifetime of the neighbouring pixel in a negative velocity direction to achieve the purpose of edge refinement. Our method directly uses the optical flow information to find the past events of the current event, and remove them to obtain the reference frame. Compared with the method of [23], our calculation is more straightforward. As the name suggests, the past events remove mechanism finds the past events of the current event and clears them. It is used to obtain a virtual frame with clear and sharp edges, which is then used as a reference frame for subsequent spatiotemporal event stream slicing. The overall description of the past events remove mechanism is shown in Algorithm 1.
**Algorithm 1** Past events remove mechanismInput: Spatiotemporal event stream: $\overset{N}{\underset{i=1}{\sum }}e\left(x_{i},y_{i},t_{i}\right)$
Output: An event stream that can form a reference frame with clear and sharp edges: $E\left(\Delta f\right)=\overset{t_{k}+\Delta f}{\underset{t_{f}}{\sum }}\{e\left(x_{i},y_{i},t_{i}\right)|t_{i}\in \left[t_{f},t_{k}+\Delta f\right]\}$
1For $e\left(x_{i},y_{i},t_{i}\right)$ in $\overset{N}{\underset{i=1}{\sum }}e\left(x_{i},y_{i},t_{i}\right)$ do2$\;R=\{e\left(x,y,t\right)|x\in \left[x_{i}-1,x_{i}+1\right],y\in \left[y_{i}-1,y_{i}+1\right],t\leq t_{i}\}$3  Calculating the optical flow information of R4  Get $\vec{v_{\left(e_{i}\right)}}$ of $e\left(x_{i},y_{i},t_{i}\right)$ by vector synthesis5  For e(x,y,t)∈R do6    2, 3, and 47  End8  Get $\vec{V_{e_{i}}}$ of $e\left(x_{i},y_{i},t_{i}\right)$ by local consistency9  Obtain the past events of $e\left(x_{i},y_{i},t_{i}\right)$ according to the movement direction of the event, and remove the past events.10End

The input of the algorithm is a spatiotemporal event stream, and the output is a spatiotemporal event stream that can form a reference frame with clear and sharp edges. The local event plane R composed of the current event and its eight neighbor events is shown in the red area in Figure 4a:

(7) $$R=\left(\begin{array}{ccc} e_{1}=\left(x_{i}-1,y_{i}+1,t_{1}\right) & e_{2}=\left(x_{i},y_{i}+1,t_{2}\right) & e_{3}=\left(x_{i}+1,y_{i}+1,t_{3}\right)\\ e_{4}=\left(x_{i}-1,y_{i},t_{4}\right) & e_{i}=\left(x_{i},y_{i},t_{i}\right) & e_{6}=\left(x_{i}+1,y_{i},t_{6}\right)\\ e_{7}=\left(x_{i}-1,y_{i}-1,t_{7}\right) & e_{8}=\left(x_{i},y_{i}-1,t_{8}\right) & e_{9}=\left(x_{i}+1,y_{i}-1,t_{9},\right) \end{array}\right)$$

This method does not use the polarity information of the event, and the polarity information does not participate in the construction of the local event plane, hence it is not displayed. If there are no eight neighbor events around the current event, the event is defined as noise and removed.

**Figure 5:**
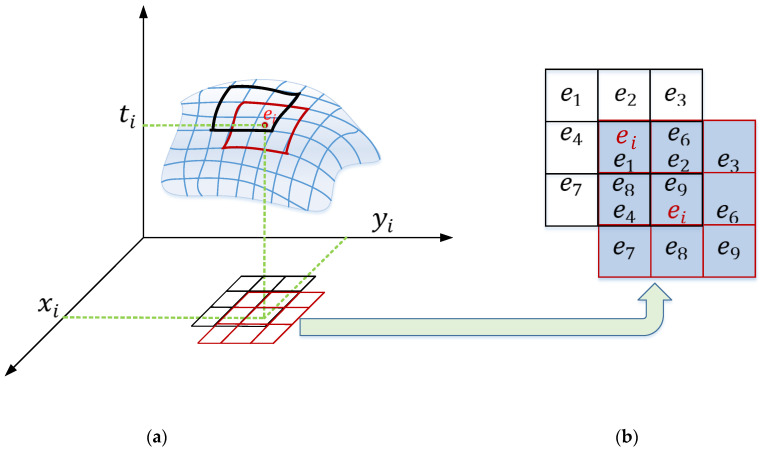
(**a**) The time surface of event; (**b**) the red and black squares in the event time surface map to the x, y plane.

Then, the optical flow of the current event and eight neighbor events is calculated by the method in [24] to synthesize the motion vector of the current event. Since a single event cannot reflect the motion information of the object, the local consistency is used so that the motion direction of the current event is determined by the motion direction of most surrounding events. Since those events in the neighborhood are triggered by the same object or pattern, the motion vectors of eight neighbor events are calculated by the same method, as shown in Figure 5b. The motion vector of the current event $\vec{V_{e_{i}}}$ is

(8) $$\vec{V_{e_{i}}}=\vec{v_{\left(e_{i}\right)}}+\overset{N}{\underset{j=1}{\sum }}\;\vec{v_{\left(e_{j}\right)}}\;\;\;\;\left(N=9,j\neq 5\right)$$

where $\;\vec{v_{\left(e\right)}}$ is the event motion vector obtained through optical flow.

**Figure 6:**
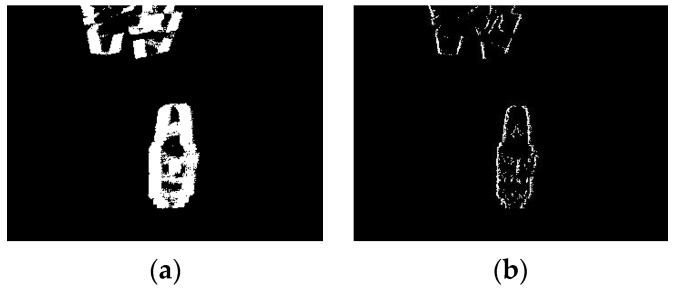
(**a**) A virtual frame composed of cumulative events; (**b**) a reference frame with clear and sharp edges obtained by the past events remove mechanism.

The past events of the current event are obtained according to the negative direction of motion of the current event, and are removed to obtain a reference frame with clear and sharp edges. According to the calculation process of the algorithm, each pixel position of our algorithm will only retain the latest time events. If multiple events occur at the same pixel position, they will be removed by the past events remove mechanism in the algorithm. As shown in Figure 6, the reference frame is defined as follows:

(9) $$\begin{array}{cc} ƒ\left(x,y,T\right) & =\overset{t_{k}+\Delta f}{\underset{t_{k}}{\sum }}\{e\left(x_{i},y_{i},t_{i},p_{i}\right)\}-\overset{t_{f}}{\underset{t_{k}}{\sum }}\{e\left(x_{i},y_{i},t_{i},p_{i}\right)\}\\ & \;\;\;\;\;\;=\overset{t_{k}+\Delta t}{\underset{t_{f}}{\sum }}\{e\left(x_{i},y_{i},t_{i},p_{i}\right)|t_{i}\in \left[t_{f},t_{k}+\Delta f\right]\} \end{array}$$

where $t_{f}$ is the start time of the event stream used to form reference frame; $t_{k}$ is the start time of the event stream used in Algorithm 1; Δf is the time length of of the event stream used in Algorithm 1; $t_{k}+\Delta f$ is the end time of the event stream used in Algorithm 1.

### 2.2. Adaptive Slicing of the Spatiotemporal Event Stream

The proposed method first calculates the similarity between the reference frame and a group of randomly constituted virtual frames in Section 2.2.1, then calculates the confidence interval of the similarity in Section 2.2.2, and adaptively updates the confidence interval in combination with the changes of the moving scene in Section 2.2.3.

If the similarity between the virtual frame formed by the accumulated events and the reference frame is within the confidence interval, it is considered that the accumulated spatiotemporal event slice contains complete object information without causing motion blur. Otherwise, it is determined whether to continue accumulating events or to update the confidence interval according to the situation of the object in the event stream. The overall description is shown in Algorithm 2.
**Algorithm 2** Adaptive slicing of spatiotemporal event streamInput: Spatiotemporal event stream: $\overset{N}{\underset{i=1}{\sum }}e\left(x_{i},y_{i},t_{i}\right)$
Output: The spatiotemporal event slice which contains complete moving object information without motion blur.$E\left(\Delta t\right)=\overset{t+\Delta t}{\underset{t}{\sum }}e\left(x_{i},y_{i},t=roundup\left(\frac{t_{i}}{1000}\right)\right)$
1Get a reference frame ƒ(x,y,T) by Algorithm 12For Δt = 1:1:n do3 $\;s_{n}\;$= Algorithm3 (E(Δt))4End5Calculate the confidence interval [α,β] of sample [$s_{1},s_{2,}\ldots \;s_{n}$]6Δt = 17For E(Δt) do8 $\;s_{n}\;$= Algorithm3 (E(Δt))9   If $\alpha <s_{n}<\beta$
10   Break. Here E(Δt) contains complete moving object information without motion blur.11  Else if ($\beta <s_{n})$ or $\left(s_{n}<\alpha \;and\;s_{n}>\;s_{n+1}\right)$
12   Break, Update confidence interval, Δt = 113  Else14   Δt=Δt+1, continue15End

#### 2.2.1. Calculation of Similarity

The spatiotemporal event stream is accumulated at an interval of 1 ms, and then the similarity is calculated with the reference frame by improved pHash [25]. The overall description is shown in Algorithm 3.
**Algorithm 3** Calculation method of image similarityInput: Spatiotemporal event stream: $E\left(\Delta t\right)=\overset{t+\Delta t}{\underset{t}{\sum }}e\left(x_{i},y_{i},t=roundup\left(\frac{t_{i}}{1000}\right)\right)$     And Output of algorithm 1: $E\left(\Delta f\right)=\overset{t_{k}+\Delta f}{\underset{t_{f}}{\sum }}\{e\left(x_{i},y_{i},t_{i}\right)|t_{i}\in \left[t_{f},t_{k}+\Delta f\right]\}$
Output: Similarity between f(x,y,T) and ƒ(x,y,T): $s_{n}$
1Build event stream E(Δt) as a virtual frame: f(x,y,T)
2Build E(Δf) as an idea virtual frame: ƒ(x,y,T)
3DCT(f(x,y,T)) and DCT(ƒ(x,y,T))
4Extract the hash values of f(x,y,T) and ƒ(x,y,T)
5Compare the similarity of hash values

Since the dynamic vision sensor only responds where the light intensity changes, its image information is high-frequency information. In order to make better use of the data characteristics of the spatiotemporal event stream, Discrete Cosine Transform (DCT) is carried out on the virtual frame.

The DCT of the virtual frame is defined as follows:

(10) $$f\left(u,v\right)=c\left(u\right)c\left(v\right)\overset{N-1}{\underset{x=0}{\sum }}\overset{N-1}{\underset{y=0}{\sum }}f\left(x,y,t\right)\cos\left[\frac{\left(x+0.5\right)\pi }{N}u]\cos[\frac{\left(y+0.5\right)\pi }{N}v\right]$$

The DCT of the reference frame is defined as follows:

(11) $$ƒ\left(u,v\right)=c\left(u\right)c\left(v\right)\overset{N-1}{\underset{x=0}{\sum }}\overset{N-1}{\underset{y=0}{\sum }}ƒ\left(x,y,T\right)\cos\left[\frac{\left(x+0.5\right)\pi }{N}u]\cos[\frac{\left(y+0.5\right)\pi }{N}v\right]$$

where c(u) is:

(12)  c(u)={1N,u=02N,u≠0

Next, we obtain the frequency coefficient matrix of the virtual frame. The frequency coefficient matrix values become higher from the upper left corner to the lower right corner. Therefore, we select the value of the 8 × 8 area in the lower right corner as a high frequency coefficient, according to experience. After that, we calculate the mean value of the high-frequency coefficients, and set the high-frequency coefficient larger than the mean value to 1 and lower than the mean value to 0 to obtain the image hash value. We compare the proportion of the hash value difference of the two images in all hash values using Hamming distance in order to obtain the similarity.

#### 2.2.2. Calculation of Confidence Interval

In order to obtain spatiotemporal event streams that contain complete object information without motion blur, we introduce the concept of the confidence interval of similarity. If the similarity between the virtual frame formed by the accumulated events and the reference frame is within the confidence interval, it is considered that the accumulated spatiotemporal event slice contains complete object information without causing motion blur.

To calculate the lower and upper bounds of the confidence interval, we collect a set of similarity $S=\left\{s_{1},s_{2},s_{3},\ldots ,s_{n}\right\}$ between the virtual frame and idea virtual frame. The mean and variance of S are $\bar{S}$ and $\delta^{2}$. Since the virtual frame is formed by the gradual accumulation of events, S are independent and distributed as a normal distribution $S\sim N\left(\mu ,\delta^{2}\right)$. Here we define a pivotal quantity *Z*, as follows:

(13) Z=S¯−μδ2n~N(0,1)

The calculation equation of confidence level 1−α is shown below:

(14) P{−Zα2≤S¯−μδ2n≤Zα2}=P{−Zα2δ2n≤S¯−μ≤Zα2δ2n}=P{S¯−Zα2δ2n≤μ≤S¯+Zα2δ2n}= 1−α

where α is a two-sided significance level. We use α=0.05 in this work, which means when the confidence level is 95%, the confidence interval of similarity *S* is obtained with the following:

(15) [α,β]=[S¯−Zα2δ2n,S¯+Zα2δ2n]

According to the t-distribution table, Zα2=1.984. In order to achieve a better slicing effect, confidence interval [α, β] always dynamically update with the moving scene, hence the sample number n will also be dynamically adjusted according to the actual scene.

#### 2.2.3. Adaptive Updating of Calculation Parameters

**Figure 7:**
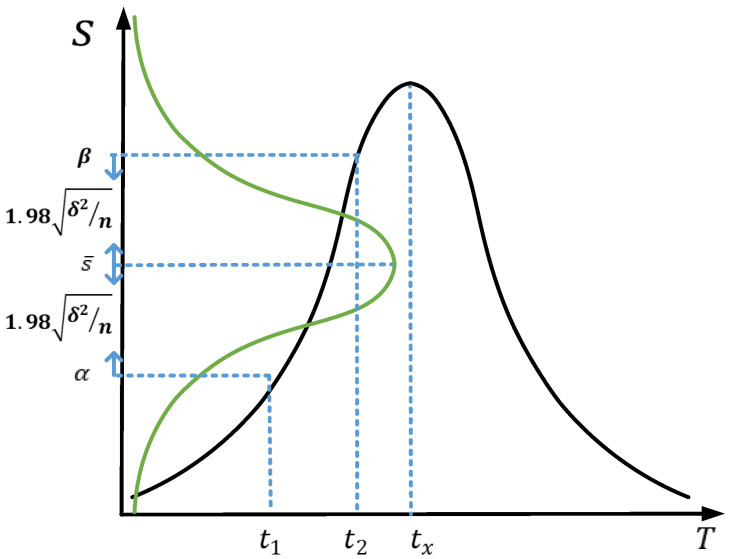
The black line represents the variation of similarity between the virtual frame and ideal frame with cumulative time; the green line is the confidence interval curve.

In theory, the similarity between the virtual frame and reference frame should meet the normal distribution with the increase of event accumulation time, as shown by the black line in Figure 7.

In order to adapt to different motion scenes, the confidence interval is updated in the following two cases:

(16) $$s_{i}=\{\begin{array}{c} s_{i}>\beta \;\;\;\;\;\;\;\;\;\;\;\;\;\;\;\;\;\;\;\;\;\;\;\;\;\;\;\;\;\;\;\;\;\;\;\;\;\;\;\;\;\;\;\;\\ s_{i}\leq \alpha \;and\;s_{i-1}>s_{i}\;\;\;\;\;\;\;\;\;\;\;\;\;\;\;\;\; \end{array}\;$$

## 3. Experiment

This section introduces the datasets used for the comparative experiment in Section 3.1, then uses four methods to carry out the comparative experiment and analyze the test results in Section 3.2.

### 3.1. Data Sets

The existing data sets play an important role in algorithm comparison. Firstly, we select the data set (Figure 7) described in [26] for comparative experiment. Then we use iniVation’s event camera DAVIS346 to build a new data set for comparative experiments in other motion scenes. It also provides a data set with appropriate complexity, and can meet different needs for scientific researchers engaged in this research. The resolution of DAVIS346 is 346 × 260 and it allows output event information (x,y,t,p), IMU data, and traditional APS frame with time information at the same time. It can meet the needs of image acquisition and index calculation of complex moving scenes.

#### 3.1.1. Public Data Sets

The data sets from [26] contains the data of objects with speed changes photographed in different scenes and different angles. The information of data sets contain:

- The asynchronous event stream;
- Intensity images at about 24 Hz;
- Inertial measurements (3-axis gyroscope and 3-axis accelerometer) at 1 kHz;
- Ground-truth camera poses from a motion-capture system k with sub-millimeter precision at 200 Hz (for the indoor data sets);
- The intrinsic camera matrix.

**Figure 8:**
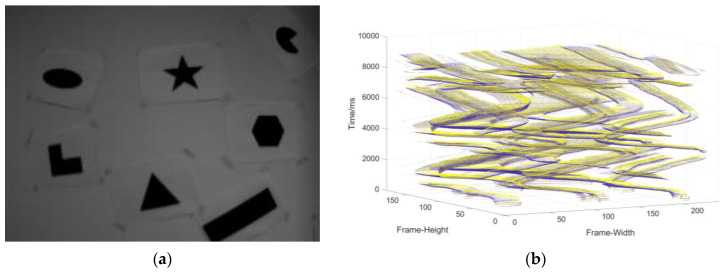
(**a**) The object used to generate the event stream; (**b**) three-dimensional spatiotemporal event stream generated by the object in (**a**).

The events, IMU data, and APS frame contained in the data sets are useful for comparison with our slicing algorithm and index calculation. Here, we only select the shapes data set from the data sets (Figure 8) for comparison.

#### 3.1.2. Our Data Sets

We use the DAVIS346 to build our data sets to verify the practical application effect of the algorithm in different motion scenes.

The data sets contain the following:

- A single moving object in a static background, such as a tank, plane, or car;
- The object having a complex motion state, such as the sudden disappearance or increase of the object in the motion scene along with a change of speed;
- Moving object in a dynamic background.

For the data sets, the motion first begins with excitation of each single degree of freedom separately; then, combined and faster excitations are performed. This results in increasing difficulty and a higher event rate over time.

The data sets contain the event stream information of the moving object, APS frame with time information, and IMU data.

**Figure 9:**
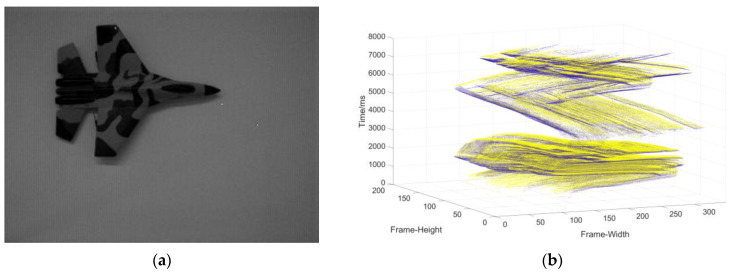
(**a**) The object used to generate the event stream; (**b**) three-dimensional spatiotemporal event stream generated by the object in (**a**).

**Figure 10:**
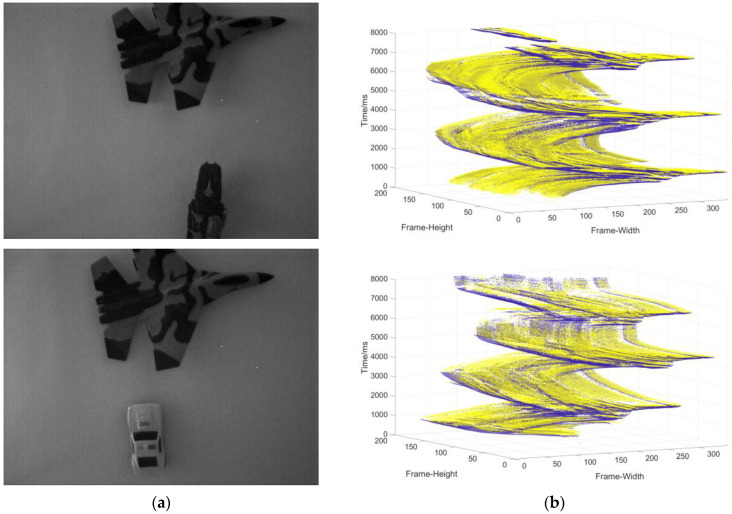
(**a**) The object used to generate the event stream; (**b**) three-dimensional spatiotemporal event stream generated by the object in (**a**).

**Figure 11:**
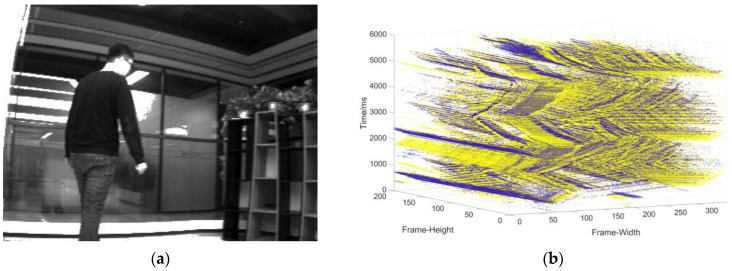
(**a**) The object used to generate the event stream; (**b**) three-dimensional spatiotemporal event stream generated by the object in (**a**).

1. Data collection of the single moving object in the static background: the camera is stationary, and the object moves at a changing speed (Figure 9).
2. Data collection in complex motion: the camera is stationary, there is multi-object motion, and the number of objects sometimes increases and sometimes decreases (Figure 10).
3. Data collection in the dynamic background: the object moves at variable speed in a complex background environment with the camera moving (Figure 11).

### 3.2. Comparisons and Analysis

We use three methods to compare with our algorithm in this paper, including constant time interval, constant event number, and ATSLTD. We take the information entropy as the comparison index. Firstly, the spatiotemporal event slicing E(Δt) is constructed into a virtual frame, and then the APS frame at the same time is found to compare the difference of information entropy between them. It is worth noting that the event camera responds to the place where the light intensity changes in the scene. Under constant external lighting conditions, only the edge and texture of the object will cause a response of the event camera. The object information in the event stream is similar to the edge information of the object. Thus, we extract the edge of the APS frame and then calculate the information entropy of the edge image for index calculation.

#### 3.2.1. Experiment I

1. Experiment

**Figure 12:**
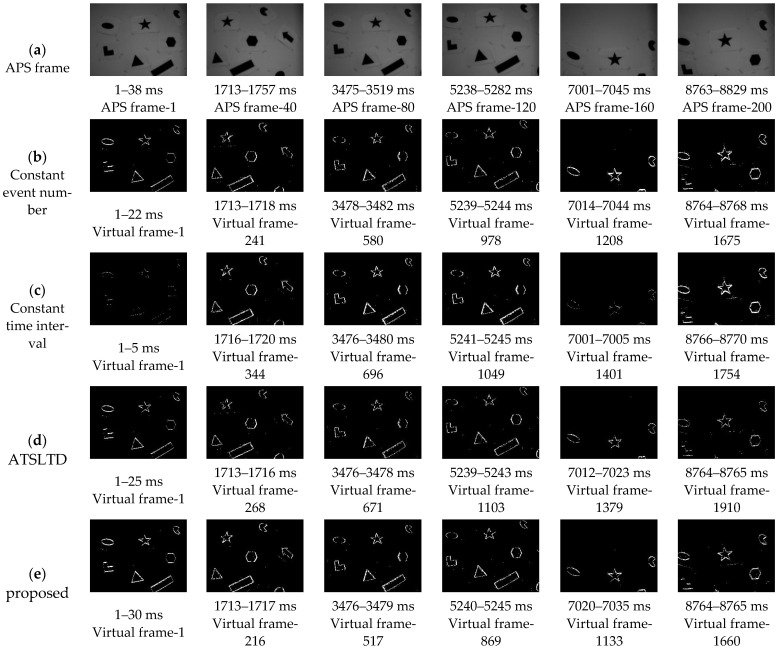
(**a**) APS frame; (**b**) virtual frame constructed with constant event number; (**c**) virtual frame constructed by constant time interval; (**d**) virtual frame constructed with ATSLTD; (**e**) virtual frame constructed with the proposed method.

In order to reflect fairness, the proposed method, the method in [21], the constant event number, and the constant time interval are used to compare the slicing effects in the public data sets [26]. Firstly, the proposed method slices the event stream in the data set into 1691 segments within 1–8828 ms. The object information contained in the event segment is neither missing nor motion blur. Secondly, the constant event number is used to slice the spatiotemporal event stream, and each slice contains 813 events (n = total number/1691); some virtual frame effect is shown in Figure 12b. Thirdly, the event stream is sliced by the method of the constant time interval, and the time length of each slice is 5 ms (Δt = total time/1691); some virtual frame effect is shown in Figure 12c. Finally, the ATSLTD is used to slice the event stream; some virtual frame effect is shown in Figure 12d. In order to judge the slicing effect more intuitively, the slicing effect pictures selected by the four methods correspond to the APS frame in Figure 12a.

2. Analysis

Visually speaking, the method of constant event number, ATSLTD, and the methods proposed achieved good results. As a result of the change of object motion speed, the event slice cut by the method of constant time interval has the phenomenon of object information loss. It is worth noting that although the slicing method with constant event number achieved a good slicing effect, the number of events selected for slicing was determined by our method. In the process of practical application, there is no possibility to obtain the number of events in advance, and the number of events can only be determined according to experience. Therefore, when the object number changes or the background changes, the constant event number cannot achieve ideal slicing effect. Since the objects in the dataset are simple geometric figures, the block information entropy will not fluctuate greatly in the process of object movement, and ATSLTD also achieved ideal results visually. However, in complex motion scenes or complex object textures, this method may not achieve ideal results. This part of the study will be carried out in experiment II and experiment III.

**Figure 13:**
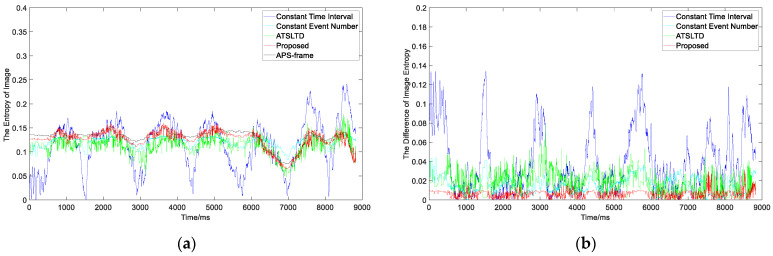
(**a**) The information entropy curve of virtual frame obtained by the four slicing methods and APS frame; (**b**) the difference curve between the information entropy of virtual frame obtained by the four methods and APS frame.

What we see is not necessarily true. In order to evaluate the slicing effect of event stream more objectively, we first construct the virtual frame with the event slice and then compare it with the information entropy of the APS frame at the same time. Figure 13a is the information entropy curve of the virtual frame obtained by four slicing methods and APS frame. Figure 12b is the difference curve between the information entropy of virtual frame obtained by four methods and APS frame. The red curve in the figure represents our proposed method. It can be seen that the difference between the information entropy of the virtual frame constructed by our proposed method and the APS frame is lower than that of the other three methods, and the difference is the smallest among the four methods. The mean value of the difference between the information entropy of the virtual frame obtained by four methods and the APS frame is shown in Table 1. It also shows that there is neither loss of object information nor motion blur in the event stream slice by the proposed method.

#### 3.2.2. Experiment II

1. Experiment

**Figure 14:**
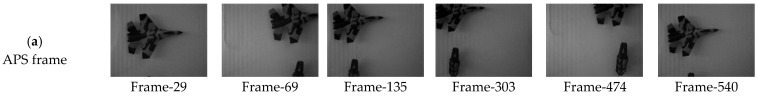

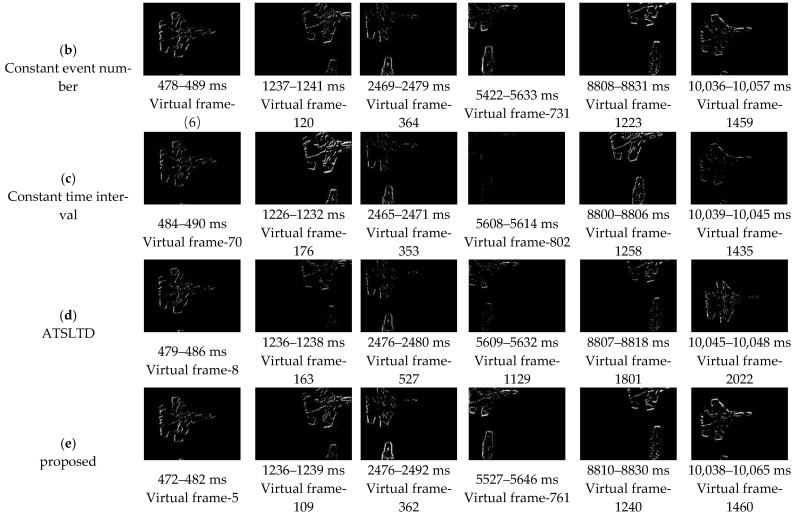
(**a**) APS frame; (**b**) virtual frame constructed with constant event number; (**c**) virtual frame constructed by constant time interval; (**d**) virtual frame constructed with ATSLTD; (**e**) virtual frame constructed with the proposed method.

Experiment 1 only verified the slicing effect on simple geometry, which is not always simple geometry in practical application. Therefore, in order to verify the slicing effect of the proposed algorithm on actual moving objects, data set (2) is selected for experiment II. Data set (2) includes Su-33 fighters with complex textures, and aircraft carriers that appear and disappear from time to time. The object’s texture and motion meet experimental requirements. The slicing effect is shown in Figure 14.

2. Analysis

**Figure 15:**
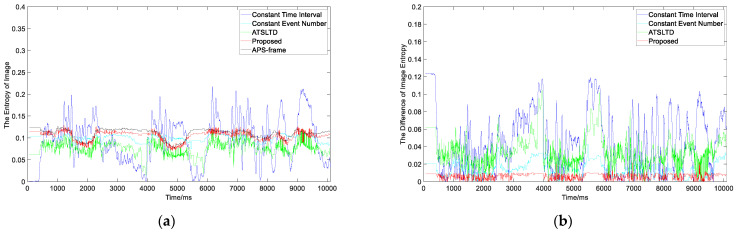
(**a**) The information entropy curve of virtual frame obtained by the four slicing methods and APS frame; (**b**) the difference curve between the information entropy of virtual frame obtained by the four methods and APS frame.

As can be seen from the slicing effect in the figure above, when the object texture is complex, and their numbers increase or decrease, the virtual frames formed by the event slice cut with our method can contain complete object information without motion blur. Other methods have more or less object information loss. Figure 15a is the information entropy curve of the virtual frame obtained by the four slicing methods and the APS frame. Figure 15b is the difference curve between the information entropy of the virtual frame obtained by the four methods and APS frame. The red curve in the figure represents our proposed method. The mean value of the information entropy difference of the proposed method is 0.0061 (Table 2), which is the smallest of the four methods. This shows that the slicing effect of the proposed method is better than that of the other algorithms, even for complex moving objects.

#### 3.2.3. Experiment III

1. Experiment

**Figure 16:**
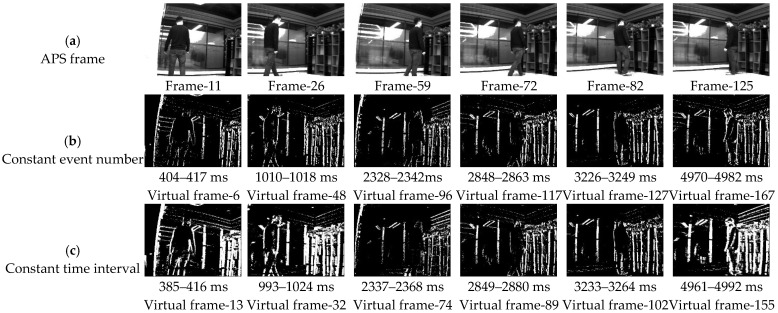

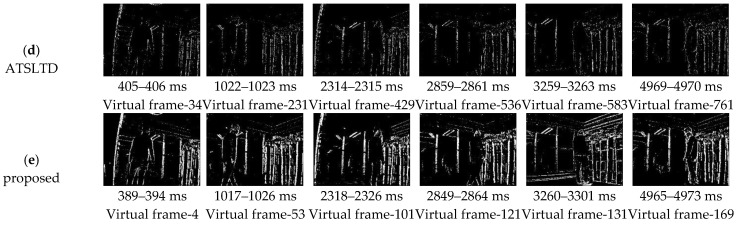
(**a**) APS frame; (**b**) virtual frame constructed with constant event number; (**c**) virtual frame constructed by constant time interval; (**d**) virtual frame constructed with ATSLTD; (**e**) virtual frame constructed with the proposed method.

Experiments I and Experiments II verify the slicing effect of the algorithm on simple geometric objects and complex moving objects using public data sets and own data sets, respectively. However, there are also moving objects in dynamic backgrounds in practical applications. This experiment aims to verify the slicing effect under dynamic backgrounds. The data set of Experiment III is data set (3), which is constructed when the object moves at variable speed in a complex background environment with the camera moving. The slicing effect is shown in Figure 16.

2. Analysis

It is worth noting that although the slicing method with the constant number of events achieved a good slicing effect, the number of events selected for slicing was determined by our method. In the process of practical application, there is no possibility to obtain the number of events in advance, and the number of events can only be determined according to experience. As a result of the changes in object speed, the object information in the event stream slice obtained with a fixed length of time has an image tail phenomenon. The confidence interval in [21] is not updated with the scene change, thus it cannot be cut effectively when the scene changes, resulting in the loss of object information. In our method, the virtual frames formed by the event stream slice in the dynamic background contain complete object information without motion blur.

**Figure 17:**
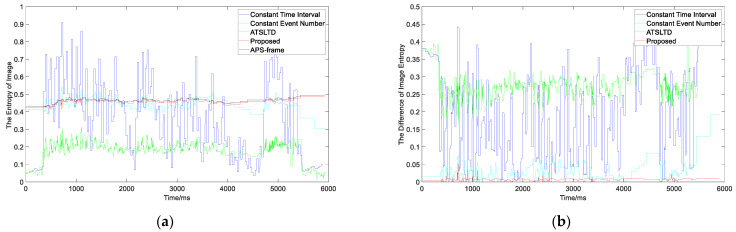
(**a**) The information entropy curve of virtual frame obtained by the four slicing methods and APS frame; (**b**) the difference curve between the information entropy of virtual frame obtained by the four methods and APS frame.

In order to evaluate the slicing effect more objectively, we compare the information entropy, as shown in Figure 17. It can be seen from the figure that the information entropy difference of our method is lower than that of the other methods, and the average value of information entropy difference is 0.0071 (Table 3).

## 4. Discussion

As a result of the above experiments, it is fully proved that the fixed number of events and fixed length of time methods exhibit the phenomenon of dragging, or lack information for the object with complex motion conditions or changing motion scenes. ATSLTD can slice effectively in a single object and simple motion scene, but there will be information loss when the motion scene is complex and changing. Our proposed method can achieve an ideal slicing effect in different motion situations, even in complex motion scenes. However, the distribution of event streams in time and space are related to the moving speed of the object. Therefore, when there are two objects with significant speed differences in the scene, our method cannot achieve a perfect slicing effect. The means of achieving a perfect slicing effect when there are two objects with significant speed differences in the scene is one of our future research directions. Moreover, the complexity of our proposed algorithm reduces the computing speed; thus, obtaining better slices with faster computing speed is another one of our future research directions. In addition, the ideal frame obtained by Algorithm 1 is used as a reference frame to slice the event stream. The ideal frame is a reference standard. Therefore, the similarity between the ideal frame of Algorithm 1 and the APS frame is higher than our algorithm, but Algorithm 1 loses events and discards the advantage of high temporal and spatial resolution of the event stream. Therefore, if one is only interested in the frame quality for computer vision processing, the output of Algorithm 1 provides valuable knowledge.

## 5. Conclusions

In this paper, we proposed an adaptive slicing method based on the spatiotemporal event stream for dynamic vision sensors, which provides a solution for the application of traditional algorithms and an appropriate preprocessing method for event-based algorithms. Each spatiotemporal event segment contains complete object information without motion blur. In order to verify the slicing effect of this algorithm in different motion scenes, this paper specially constructed the data sets, and provided a data set with appropriate complexity to meet different needs for scientific researchers engaged in this field. The proposed method was compared with other methods using different data sets. The results showed that the difference between the information entropy of the virtual frame and the APS frame is lower than for other methods. This method is not only suitable for various complex motion scenes, but also better than existing algorithms.

## Figures and Tables

**Table 1:** The mean value of the difference between the information entropy of virtual frame obtained by the four methods and APS frame.

The Method	The Mean of Average Difference
Constant Event Number	0.0150
Constant Time Interval	0.0390
ATSLTD	0.0186
Proposed	0.0064

**Table 2:** The mean value of the difference between the information entropy of virtual frame obtained by the four methods and APS frame.

The Method	The Mean of Average Difference
Constant Event Number	0.0135
Constant Time Interval	0.0459
ATSLTD	0.0335
Proposed	0.0061

**Table 3:** The mean value of the difference between the information entropy of virtual frame obtained by the four methods and APS frame.

The Method	The Mean of Average Difference
Constant Event Number	0.0393
Constant Time Interval	0.2064
ATSLTD	0.2884
Proposed	0.0071

## Data Availability

Not applicable.
